# Transcriptional Regulation of Small Heat Shock Protein 17 (sHSP-17) by *Triticum aestivum HSFA2h* Transcription Factor Confers Tolerance in *Arabidopsis* under Heat Stress

**DOI:** 10.3390/plants12203598

**Published:** 2023-10-17

**Authors:** Ranjeet R. Kumar, Kavita Dubey, Suneha Goswami, Gyanendra K. Rai, Pradeep K. Rai, Romesh K. Salgotra, Suman Bakshi, Dwijesh Mishra, Gyan P. Mishra, Viswanathan Chinnusamy

**Affiliations:** 1 Division of Biochemistry, Indian Agricultural Research Institute, New Delhi 110012, India; dubeykavita786@gmail.com; 2 School of Biotechnology, Sher-e-Kashmir University of Agricultural University of Jammu (J&K), Jammu 180009, India; gkrai75@gmail.com (G.K.R.); pradeepr2000@gmail.com (P.K.R.); schoolofbiotechnology@gmail.com (R.K.S.); 3 Nuclear Agriculture & Biotechnology Division, Bhabha Atomic Research Center, Trombay, Mumbai 400085, India; sood004@gmail.com; 4 Centre for Agricultural Bio-Informatics, Indian Agricultural Statistics Research Institute, New Delhi 110012, India; dwij.mishra@gmail.com; 5 Division of Seed Technology, Indian Agricultural Research Institute, New Delhi 110012, India; gyan.gene@gmail.com; 6 Division of Plant Physiology, Indian Agricultural Research Institute, New Delhi 110012, India; viswa.chinnusamy@gmail.com

**Keywords:** heat shock transcription factor, heat stress, small HSP, wheat, total antioxidant potential, GPX, CAT, SOD

## Abstract

Heat shock transcription factors (HSFs) contribute significantly to thermotolerance acclimation. Here, we identified and cloned a putative HSF gene (*HSFA2h*) of 1218 nucleotide (acc. no. KP257297.1) from wheat *cv.* HD2985 using a de novo transcriptomic approach and predicted sHSP as its potential target. The expression of *HSFA2h* and its target gene (*HSP17*) was observed at the maximum level in leaf tissue under heat stress (HS), as compared to the control. The *HSFA2h*-pRI101 binary construct was mobilized in Arabidopsis, and further screening of T3 transgenic lines showed improved tolerance at an HS of 38 °C compared with wild type (WT). The expression of *HSFA2h* was observed to be 2.9- to 3.7-fold higher in different Arabidopsis transgenic lines under HS. *HSFA2h* and its target gene transcripts (*HSP18.2* in the case of Arabidopsis) were observed to be abundant in transgenic Arabidopsis plants under HS. We observed a positive correlation between the expression of *HSFA2h* and *HSP18.2* under HS. Evaluation of transgenic lines using different physio-biochemical traits linked with thermotolerance showed better performance of HS-treated transgenic Arabidopsis plants compared with WT. There is a need to further characterize the gene regulatory network (GRN) of *HSFA2h* and sHSP in order to modulate the HS tolerance of wheat and other agriculturally important crops.

## 1. Introduction

Plants are exposed to various abiotic stresses, mainly salinity, drought, high and low temperatures, chemical pollutants, etc., which adversely affect their growth and development. Heat stress (HS) is one of the major problems among the various abiotic stresses reported to date [1]. Heat stress causes outbursts of reactive oxygen species (ROS) and inactivation/denaturation of many heat-labile proteins and enzymes, which ultimately affect the growth, development, and reproduction of plants [2,3]. Heat stress has been reported to affect the pollen–stigma interaction and cause drying of stigmatic fluid, improper fertilization, and defragmentation of starch granule synthesis, which further culminates in the formation of shriveled grains with low test weight [4]. Plants have inherited defense mechanisms to cope with the vagaries of nature. Various physiochemical and molecular mechanisms operate inside the plant system in order to modulate the tolerance level in response to different biotic and abiotic stresses. The expression of stress-associated genes (SAGs)/proteins (SAPs) in response to HS is regulated by various heat-responsive transcription factors (*HSFs*) [5,6]. HSFs play a very important role in regulating the expression of genes linked with tolerance against abiotic stresses [7,8]. The functionality of HSFs against HS has been reported in many agriculturally important crop plants [1,9]. 

HSF genes have been reported from different crop plants like rice (25), Arabidopsis (21), wheat (56), etc., and have been classified into three classes: *HSFA*, *HSFB*, and *HSFC* [10,11]. Among HSF members, *HSFA* genes were observed to bind to heat shock elements (HSEs) and have been functionally validated in Arabidopsis. Many HSFs have even been reported to express constitutively under non-stress conditions. The accumulation of other HSFs, especially A2 and A6 members, was reported to increase under HS. Abiotic stressors, especially drought and salt, have been reported to increase the expression of HSFs many-fold in different plant species [11]. Plant HSFs contribute significantly to the networks involved in stress signaling and stress response and have a very conserved structure. *HSFA1a* identified from tomato showed regulatory association with *HSP90*/*HSP70* and was observed to maintain an inactive monomer state under control conditions and form an active trimer in response to HS upon dissociation [12]. The expressions of *HSFA1a* and *HSFA1b* were observed constitutively in Arabidopsis and are considered early response factors for stress [13,14]. They mainly serve as transcriptional activators for other HSF members, such as *HSFA2*, and play a very important role in acquired HS tolerance [15]. Mostly, *HSFA* subclasses—A1, A2, A3, A4, and A9—function as transcriptional activators for *HSP* genes [16,17], whereas *HSFA5* acts as a repressor for *HSFA4* [18]. The main characteristic of HSF proteins is the presence of a DNA-binding domain through which they bind to HSEs with a consensus sequence of GAAnnTTCnnGAA [19,20]. HSEs have been reported to be present in the promoters of many HSP genes. Several HSFA proteins have the potential to bind to HSE sequences, and each subclass of HSFA proteins regulates a subset of HS-responsive genes [21,22,23]. HSF proteins regulate the expression of HSP genes and, in turn, modulate the HS-tolerance level of plants [6,12,24].

There is a correlation between oxidative stress, the HS response, and the modulation of HS tolerance. Panchuk et al. [25] studied the effect of HS on the activity and expression of antioxidant enzymes like superoxide dismutase (SOD), catalase, ascorbate peroxidase (APX), etc. HSF3 overexpressing transgenic Arabidopsis plants showed higher APX transcript accumulation and an increase in APX activity. Other reports also showed the connection between overexpressing HSFs (like HSFA2 and *At*HSFA8) and oxidative stress response in Arabidopsis and tomato [26]. 

HSFs reported in plants have been observed to play a dual role in regulating the expression of SAGs as well as regulating the growth and development, cell proliferation, and differentiation of the plant. *AtHSFA2* gene expression was found to be increased during callus formation and growth from root explants [27]. Similarly, overexpression of *OsHSFA2a* in response to HS was observed to increase the growth and development of panicles and seeds in rice [19]. The expression of *OsHSFA7* and *A9* was observed to be higher in the developing seeds of rice; a similar pattern of expression of *HSFA9* was observed in the case of sunflower and *Arabidopsis* [12,28].

Here, we have identified and cloned a putative heat shock TF gene (*HSFA2h*) from wheat and further established its role in modulating the HS-tolerance level of the plant through functional validation with Arabidopsis.

## 2. Results

### 2.1. Identification of Transcripts Predicted to Be HS-Responsive Transcription Factor (HSF)

The transcriptome data generated in our lab (BioProject acc. no PRJNA171754) were mined for the prediction of transcripts coding for HSF. Mining of the annotated data based on the domain search (presence of DNA binding domain; Table S1) revealed the presence of 37 putative HSF transcripts. Based on the digital gene expression (DGE) analysis, we targeted transcript_23590 for cloning and characterization. 

### 2.2. Cloning and In Silico Characterization of Putative HSFA2h

**Figure 1:**
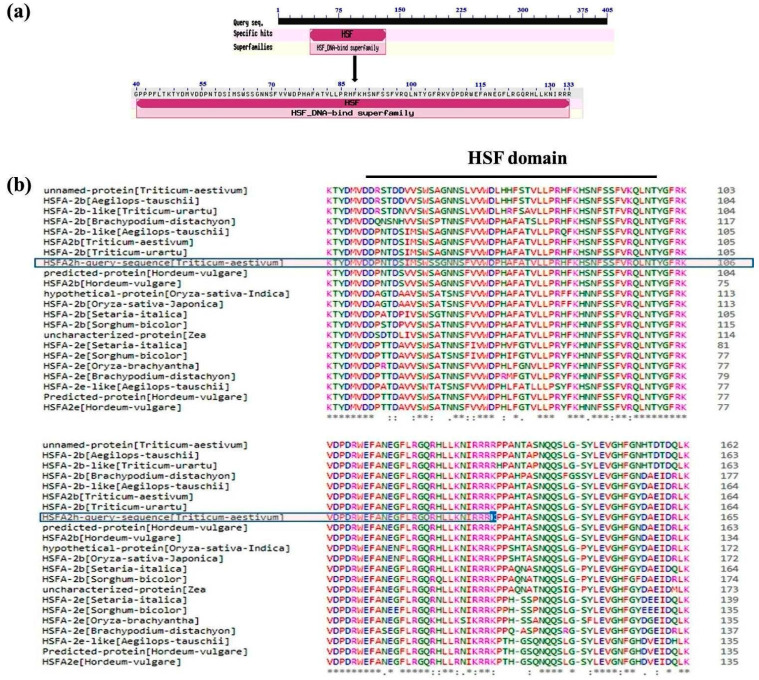
Domain organization of HSFA2h and comparison with its orthologue proteins in other plant species: (**a**) schematic representation of DNA binding domains in HSFA2h protein by CD search tool, (**b**) full-length protein alignment of HSFA2h with its orthologue by Clustalx2.

An amplicon of ~1.2 kb was amplified from wheat cv. HD2985 using transcript-specific primers. The amplified product was purified and cloned in a pGEM-T Easy vector. Sanger’s sequencing using the di-deoxy method showed the presence of 1218 nucleotides with an open reading frame of 405 aa. Based on the homology search, the gene was named HSFA2h. The nucleotide sequence was submitted to NCBI (GenBank acc. no. KP257297.1). A BLASTn search showed maximum homology with HSFA2h reported from Triticum (acc. no. KF208545.1) and a cDNA clone (NIASHv1090C03) from *Hordeum vulgare* (acc. no. AK359122.1). A protein-based homology search showed maximum (99%) homology with an HSFA2h gene reported from Triticum aestivum (acc. no. AHZ44766.1) followed by 90% with an HSFA2b gene reported from Triticum aestivum (acc. no. AHZ44765.1) and 89% with HSFA-2b-like from Aegilops tauschii subsp. tauschii (acc. no. XP_020146162.1). A prominent HSF domain was observed between the regions 40 and 133 aa containing the sequence-specific DNA binding domain and sequence-specific DNA binding transcription factor activity (Figure 1). 

A phylogenetic analysis of cloned HSFA2h represents its close relationship with HSFA2b of Hordeum vulgare, Aegilops tauschii subsp. tauschii and Triticum aestivum (Figure S1). Small HSPs, such as HSP18.1, HSP18.2, HSP22, etc., were predicted to be the target genes of HSFA2h by the Nsite program (www.softberry.com) (accessed on 22 February 2023). 

### 2.3. Expression Analysis of Cloned HSFA2h TF and Its Target Gene in Wheat under HS

We analyzed the tissue-specific expression of HSFA2h TF during pollination and grain-filling stages in contrasting wheat cvs. under HS. Since small HSPs were predicted to be the targets of HSFA2h, we targeted the HSP17 gene (cloned in our lab from wheat) showing ~99% homology with the HSP18.2 gene reported from Arabidopsis thaliana for the correlation study.

**Figure 2:**
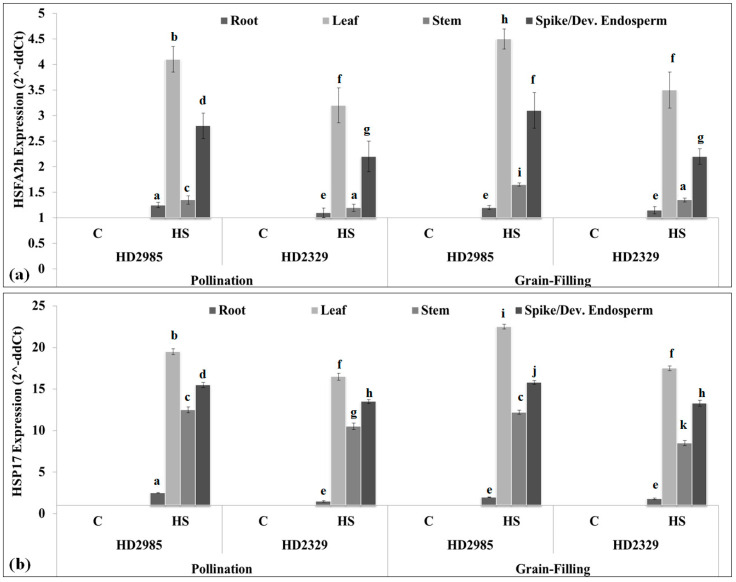
Expression analysis of HSFA2h TF and its target gene in contrasting wheat cvs. under heat stress: (**a**) expression of HSfA2h during pollination and grain-filling stages, (**b**) expression of HSP17 during pollination and grain-filling stages; C—control (22 ± 3 °C), HS—38 °C, 1 h; all data are presented as mean ± SE of three replicates, and different letters above each bar indicate a significant difference between treatments (*p* ≤ 0.05, one-way ANOVA).

During the pollination stage, the expression of HSFA2h in wheat cv. HD2985 was observed to be the maximum in leaf tissue (4.1-fold) under HS, followed by a spike (2.7-fold), as compared to control (Figure 2a). A similar pattern of expression was observed in the case of thermosusceptible wheat cv. HD2329 under HS, though the abundance of the transcript was observed to be less as compared to thermotolerant cv. HD2985.

During the grain-filling stage, the HSFA2h expression in wheat cv. HD2985 was observed to be the maximum (4.5-fold) in the HS-treated leaf, whereas it was observed to be the minimum (1.25-fold) in root tissue (Figure 2a). We observed an abundance of the transcript in the stem of wheat cv. HD2985 under HS as compared to the stem of wheat cv. HD2329. Overall, the transcripts of HSFA2h under HS were observed to be the maximum in the leaf tissue, followed by spike and stem, and the minimum in the root; thermotolerant cv. HD2985 showed higher expression as compared to thermosusceptible wheat cv. HD2329.

Expression of sHSP17 (target of HSFA2h) during the pollination stage showed the maximum transcripts in the leaf of wheat cv. HD2985 (19.5-fold), followed by the spike and stem under HS (Figure 2b). A similar pattern of HSP17 expression was observed in wheat cv. HD2329 during the pollination stage. Expression analysis during grain-filling showed maximum expression in the leaf of wheat cv. HD2985 (23.5-fold) under HS, followed by the spike and stem. Similarly, wheat cv. HD2329 showed maximum expression of HSP17 in the leaf (16.5-fold), followed by the spike and stem under HS. We established a positive relationship between the expression of HSFA2h and HSP17 in different tissues of wheat under HS. 

### 2.4. Validation of Transgenic Arabidopsis Plants Overexpressing Wheat HSFA2h Transcription Factor

**Figure 3:**
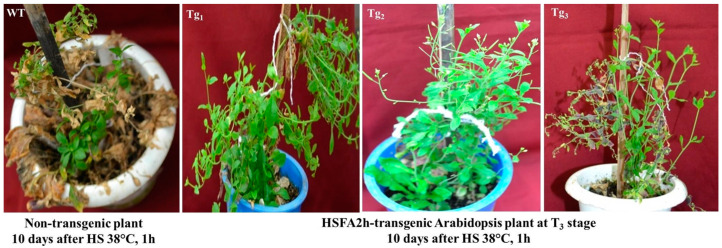
Phenotypic observation of HSFA2h TF expressing transgenic Arabidopsis plants (at T3 stage) along with wild-type plants exposed to heat stress of 38 °C for 1 h.

The binary construct of wheat HSFA2h was developed in the pRI101-AN vector under the 35S promoter (Figure S2). Transformation of the pRI101-HSFA2h construct in Arabidopsis thaliana (ecotype Columbia) was attempted through the floral dip method. Three independent transgenic Arabidopsis lines were developed and forwarded to the T_3_ stage, where they were checked for thermotolerance by subjecting them to HS of 38 °C for 1 h at the inflorescence stage. Wild-type (WT) plants were also given the same heat treatment, and plants were kept in regulated conditions inside the National Phytotron Facility, ICAR-IARI, New Delhi, for further observation. WT plants gradually turned brown, while transgenic Arabidopsis plants showed improved thermotolerance and seed setting (Figure 3). 

**Figure 4:**
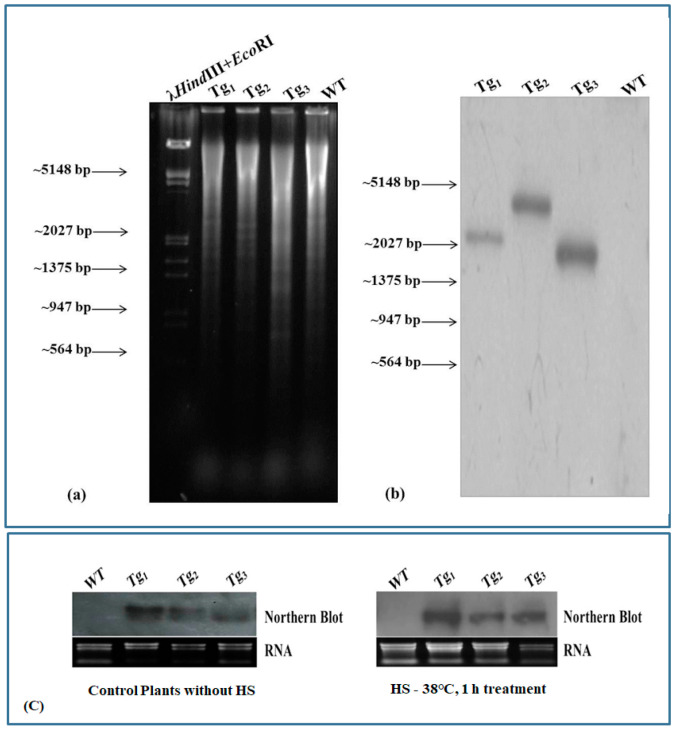
Southern and Northern blot analysis of transgenic Arabidopsis harboring wheat HSFA2h TF gene: (**a**) restriction analysis of isolated genomic DNA, (**b**) blot developed after probing with TaHSFA2h TF, (**c**) Northern blot analysis; WT—wild type Arabidopsis, Transgenic Arabidopsis lines—Tg_1_, Tg_2_, and Tg_3_ showing the integration of wheat HSFA2h TF gene; plants were exposed to HS of 38 °C, 1 h.

Transgenic plants harboring the wheat HSFA2h transcription factor were confirmed by Southern hybridization. We observed a single prominent blot in each lane restricted by NotI (Figure 4a,b). 

This result suggests that the cloned HSFA2h gene has been integrated into the Arabidopsis plant in a single copy number. Further, the expression of HSFA2h was also checked by Northern blot hybridization. Expression of HSFA2h was observed to be higher in transgenic Arabidopsis plants at the T_3_ stage through Northern blot hybridization, while wild-type plants did not show any expression. The expression of HSFA2h was observed to be higher under HS as compared to control plants (Figure 4c).

### 2.5. Expression Analysis of HSFA2h and Its Target Gene (HSP 18.2) in Transgenic Arabidopsis Exposed to HS at T_3_ Stage

**Figure 5:**
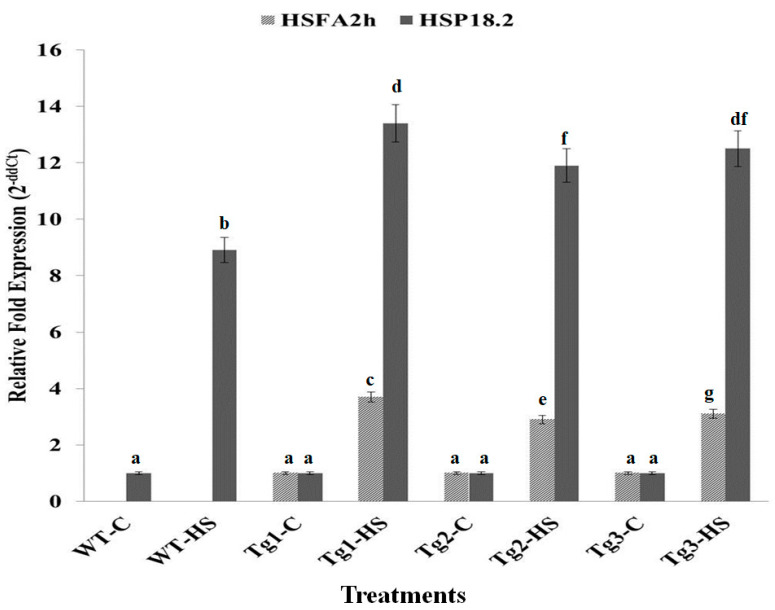
Expression analysis of *HSFA2h* TF and its target gene *HSP18.2* through quantitative real-time PCR in WT (wild type) and transgenic Arabidopsis lines; Tg_1_, Tg_2_, and Tg_3_—three different transgenic lines, C—control (22 ± 3°), HS—heat stress (38 °C for 1 h); all data are presented as mean ± SE of three replicates, and different letters above each bar indicate a significant difference between treatments (*p* ≤ 0.05, one-way ANOVA).

In silico analysis by the Nsite program showed that sHSPs are probable targets of HSFA2h. The primers for Arabidopsis HSP18.2 (acc. no. NM_125364.3) were designed for qRT-PCR. The transcript accumulation of HSFA2h and their target gene, i.e., HSP18.2, in transgenic Arabidopsis plants at the T_3_ stage showed many-fold increases in their expression in response to HS of 38 °C for 1 h as compared to control (Figure 5). 

The expression of HSFA2h in transgenic Arabidopsis was found to be 2.9- to 3.7-fold higher under HS as compared to control transgenic plants. Wild-type plants did not show any expression for HSFA2h, but the expression of HSP18.2 was 8.9-fold higher under HS as compared to the WT control plant. The expression of HSP18.2 was higher in transgenic lines (11.9- to 13.4-fold) as compared to WT (8.9-fold). We observed a positive correlation between the expression of HSFA2h and HSP18.2 under HS. The expression analysis of HSFA2h and HSP18.2 in transgenic Arabidopsis and their phenotypic observation after HS treatment suggest that HSFA2h plays a significant role in modulating HS tolerance by regulating the expression of HSP18.2. 

### 2.6. Biochemical Screening of Transgenic Arabidopsis under HS

**Figure 6:**
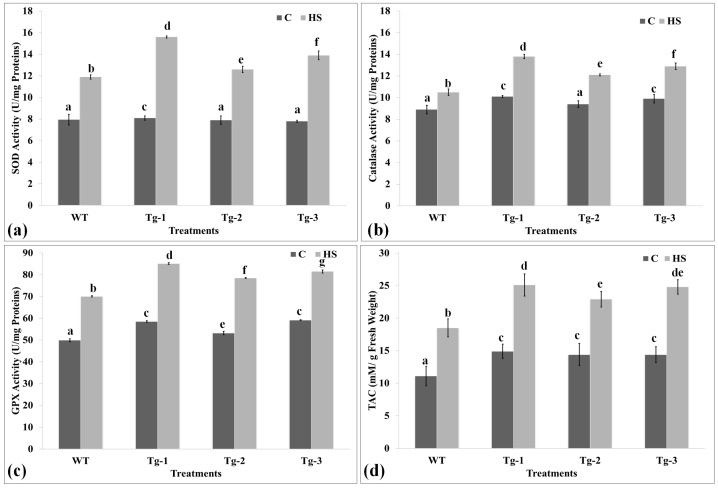
Biochemical analysis of *HSFA2h* TF-expressing Arabidopsis transgenic lines at T3 stage for their heat stress tolerance level: (**a**) superoxide dismutase (SOD) activity assay, (**b**) catalase (CAT) activity assay, (**c**) guaiacol peroxidase (GPX) activity assay, and (**d**) total antioxidant capacity (TAC) assay; WT—wild type, Arabidopsis transgenic lines—Tg_1_, Tg_2_, and Tg_3_, C—control, HS—heat stress of 38 °C for 1 h; all data are presented as mean ± SE of three replicates, and different letters above each bar indicate a significant difference between treatments (*p* ≤ 0.05, one-way ANOVA).

Antioxidant enzymes metabolize the toxic free radicals produced due to stress and help the plants combat unfavorable environments. The samples collected from WT and transgenic plants were analyzed for their thermotolerance level through an activity assay of antioxidant enzymes (Figure 6). 

The SOD activity assay was observed to be the maximum (15.5 U/mg protein) in Tg-1 under HS, followed by Tg-3 (13.8 U/mg protein), whereas it was observed to be the minimum (7.9 U/mg protein) in WT under control conditions (Figure 6a). HS-treated transgenics showed significantly higher SOD activity as compared to the control samples. 

The catalase enzyme acts on the superoxide radicals and helps to neutralize them inside the cells. Catalase showed the maximum activity (13.7 U/mg proteins) in Tg-1 under HS, whereas the minimum activity (8.6 U/mg proteins) was observed in WT under control conditions (Figure 6b). GPx activity showed a similar pattern of activity in transgenic under HS as compared to the control (Figure 6c). Overall, the antioxidant enzymes’ activity was observed to be maximum in transgenic lines under HS as compared to WT and other control samples. 

The total antioxidant potential is an indirect method to assay the thermotolerance of a plant. We observed an increase in the total antioxidant potential of transgenic Arabidopsis plants under HS as compared to WT (Figure 6d). The maximum TAC (25.1 mM/g FW) was observed in Tg-1 under HS, followed by Tg-3, whereas the minimum (11.1 mM/g FW) was observed in WT under control conditions. All three transgenic lines showed comparatively higher total antioxidant potential compared with WT. 

### 2.7. Alterations in the Photosynthesis-Associated Parameters under HS

**Figure 7:**
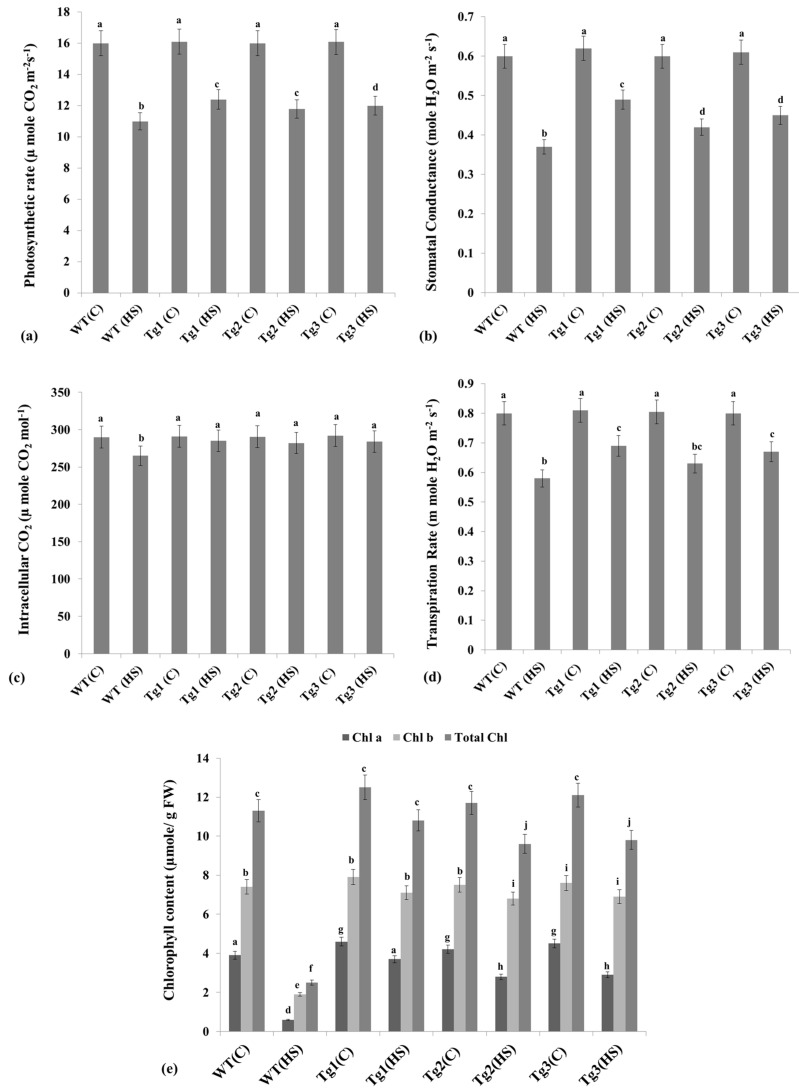
Alterations in the photosynthesis-associated parameters in WT and transgenic lines of Arabidopsis under HS: (**a**) photosynthetic rate, (**b**) stomatal conductance, (**c**) intracellular carbon dioxide, (**d**) transpiration rate, (**e**) chlorophyll content; C—control, HS—heat stress (38 °C, 1 h); all data are presented as mean ± SE of three replicates, and different letters above each bar indicate a significant difference between treatments (*p* ≤ 0.05, one-way ANOVA).

We observed a significant decrease in the photosynthetic rate in response to HS (38 °C, 1 h) in both WT plants and HSFA2h-overexpressing transgenic lines. A percent decrease in the photosynthetic rate was observed to be the minimum in the Tg-1 transgenic line as compared to the WT under HS (Figure 7a). 

Stomatal conductance was also decreased due to HS in all the plants; however, a greater percent decrease was observed in WT than in the transgenic lines (Figure 7b). A significant decrease in intracellular CO_2_ was observed in response to HS; differences were, however, non-significant in transgenic lines compared to WT plants (Figure 7c). Similarly, the transpiration rate also decreased due to HS, but the decrease was observed at its maximum in WT rather than in the transgenic lines (Figure 7d). 

We also analyzed the effect of HS on Chl content (Chl a, Chl b, total Chl) in WT and transgenic lines. Chl a was observed at the maximum (4.6 µmol/g FW) in Tg-1 under control conditions, whereas it was observed at the minimum (0.45 µmol/g FW) in WT under HS conditions (Figure 7e). A similar pattern of Chl b and total Chl was observed in response to HS. A percent decrease in Chl a, Chl b, and total Chl under HS was observed at the minimum in the Tg-1 transgenic line, whereas it was observed at the maximum in the WT. The WT plants showed a faster depletion of photosynthetic pigments than the HSFA2h-overexpressing transgenic plants. After 10 days of exposure to HS, HSFA2h-overexpressing transgenic lines still maintained a stable total chlorophyll content, whereas WT plants were almost completely devoid of chlorophyll.

## 3. Discussion

Heat stress has always been considered one of the major abiotic stresses affecting the growth, yield, and quality of grains [29]. Various mechanisms and regulatory networks operate inside the plant system in order to cope with HS. Heat-responsive TFs play a very important role in driving the SAGs, especially heat shock proteins (HSPs), involved in modulating the tolerance of the plants [4]. HSPs play a dual role in protecting the nascent proteins from denaturation/aggregation under HS, with a few of them acting as signaling molecules that trigger the defense network of the plants [5,6]. Guo et al. [29] identified ~82 HSF members in a genome-wide study on wheat (*Triticum aestivum* L.). In the present investigation, we cloned ~1.2 kb HSF TF from wheat belonging to the A2h family. Saidi et al. [30] identified >15 HSFs in wheat through transcriptome meta-analysis and validated the candidate TFs to play a very important role in the broad stress tolerance in wheat. In silico characterization of the cloned *HSFA2h* showed the presence of a sequence-specific DNA binding domain, which is characteristic of any transcription factor [9]. We identified small HSPs as the probable targets of *HSFA2h* TF. Schramm et al. [16] reported the direct binding of HSFA2 to the promoters of small HSPs such as HSP26.5, HSP22, HSP25.3, HSP18.1, etc. Several studies reported that HSFs regulate the expression of stress-responsive genes such as HSPs under several abiotic stresses [2,5]. The DNA-binding domain of wheat HSF also binds to the functional HSE sequences present in the promoter regions of HSP genes [11]. Plant HSFs are the essential components of a signal transduction chain, regulating the expression of genes involved in various abiotic stresses, especially HS [7]. Xue et al. [11] reported that the transcript levels of HSFA2 and HSFA6 members predominate under HS in wheat. Ma et al. (2023) observed an increase in the expression of 14 HSFs including HSFA2h in drought-primed seeds in wheat under heat stress. To validate the potential of the wheat HSFA2h gene, it was transformed in Arabidopsis plants, and transgenic plants harboring wheat HSFA2h were confirmed by Southern and Northern blotting. The Southern positive transgenic plants at the T3 stage were subjected to HS treatment of 38 °C for 1 h, and plants were kept under regular observation. We observed an increase in the transcript accumulation of HSFA2h and its target gene, i.e., HSP18.2, in transgenic Arabidopsis under HS as compared to control. Meena et al. [4] reported that TaHsfA6b-4D plays a very important role in linking HSR with the unfolded protein response and helps in maintaining protein homeostasis under heat stress. Ma et al. [31] reported that alternative splicing of TaHsfA2-7 causes increase in the thermotolerance level of wheat. Wen et al. [32] observed that HSP-mediated translational regulation was enhanced in response to alternate splicing of TaHSFA6e in wheat under HS. The relevant biochemical parameters such as TAC, SOD, CAT, and GPX analysis also showed significant results, which conclude that HSFA2h plays an important regulatory role in the development of tolerance against HS by modulating the expression of HSPs and genes associated with antioxidant enzymes. It was also observed that non-transgenic plants do not tolerate HS and gradually turn brown due to the destruction of the chlorophyll content, leading to an alteration in photosynthesis-associated parameters like photosynthetic rate, stomatal conductance, intracellular CO_2_, and transpiration rate, while transgenic Arabidopsis plants showed better photosynthetic performance and hence conferred an improved tolerance to high-temperature stress. Tian et al. [33] reported that HSF A1b regulates HS tolerance in Arabidopsis and wheat through OPR3 and jasmonate signaling pathways. Several HSPs function as molecular chaperones, which are capable of stabilizing thermo-labile proteins against heat denaturation [3,34]. For example, chloroplast photosystem II electron transport activity is protected from heat inactivation by a small HSP in the chloroplast [35]. Similarly, at high temperatures, the electron transport activity of the mitochondrial NADH: ubiquinone oxidoreductase complex I in plants is enhanced by a mitochondrial small HSP [35]. Wan et al. [36] reported that overexpression of a class III sHSP gene (PmHSP17.9) in *Prunus mume* modulates the HS-tolerance level of transgenic Arabidopsis by enhancing superoxide dismutase (SOD) activity. The above observations emphasize that HSFA2h and its target gene, i.e., HSP18.2, play a major role in developing thermotolerance and that they may be considered as potential markers for evaluating the diverse germplasm of wheat for the development of a ‘climate-smart’ crop.

## 4. Materials and Methods

### 4.1. Plant Material and Stress Treatment

Seeds of two popular wheat *cvs*., HD2985 (thermotolerant) and HD2329 (thermosusceptible), were procured from the Division of Genetics, Indian Agricultural Research Institute (IARI), New Delhi. Seeds pre-treated with Bavistin at 0.25% were sown in pots (12 inches) inside climate-regulated chambers with day/night temperatures of 22/18 °C, RH of 75%, and light intensity of 250 μmol^−2^S^−1^. Mixture of perlite, FYM, and fine sand was used for the pot-filling. Twelve pots were divided into two groups: one set for the control (22 ± 2 °C) and another set for the HS treatment (38 °C, 1 h) in triplicate. Plants were exposed to HS inside microprocessor-regulated chambers in a sinusoidal mode with an increment of 1 °C every 10 min until they reached the HS-treated temperature [6]. After HS, the temperature was lowered to the ambient temperature in the same fashion. Samples of leaf, stem, and developing endosperm were collected in triplicate at pollination and grain-filling stages, as per the Feekes scale [37], frozen in liquid nitrogen, and stored at −80 °C for further downstream analysis.

The *A. thaliana* wild-type and transgenic plants used in the present investigation were from the Columbia genetic background. The seeds were sown in groups of two [Group-I: control (day-time: 25 ± 3 °C, night-time: 18 ± 3 °C), Group-II: (day-time: 36 ± 3 °C, night-time: 30 ± 3 °C)] inside microprocessor regulated growth chamber at National Phytotron facility, ICAR-IARI, New Delhi. The chambers were exposed to 16/8 h light/dark cycle with a light intensity of 120–150 µmol/m^2^. Other steps for growing the plants were followed, as mentioned by Clough et al. [38].

### 4.2. RNA-Seq for the Identification of HSF Transcripts

The data generated from the de novo transcriptome sequencing of control and HS-treated samples of wheat *cvs*. HD2985 and HD2329 in our lab (BioProject accession no. PRJNA171754) were used for mining the heat-responsive genes/transcription factors. The transcript sequences coding for the protein with heat-responsive transcription factor-specific domains were mined. We observed 37 different transcripts with DNA-binding domains showing homology with HSFs reported from other plant species. The predicted HSF transcripts, which showed maximum digital fold expression, were used for further cloning and characterization.

### 4.3. Molecular Cloning of Wheat HSF Gene

#### 4.3.1. Transcript-Specific Oligo Designing

The transcript predicted to be HSF was used for the primer design using Genefisher 2 Primer designing software (https://bibiserv.cebitec.uni-bielefeld.de/genefisher2) (accessed on 27 February 2023), applying all the default parameters like length of primer at 18–22 bases, Tm between 58 and 60 °C and it should not form secondary structure. The primers were subjected to quality checks using OligoCalc (http://www.basic.northwestern.edu/biotools/oligocalc.html, accessed on 27 February 2023); HPLC-purified oligos were synthesized commercially (Table 1).

#### 4.3.2. Isolation of Total RNA and RT-PCR Amplification of Gene

Total RNA was isolated from the control and HS-treated leaf samples of wheat *cvs*. HD2985 and HD2329 by the Trizol method (Invitrogen, Carlsbad, 5781 Van Allen Way, UK). The quality of the RNA was checked using Bioanalyzer (Agilent, Cheadle, UK); RNA samples with OD 260/280 ratio of more than 2.0 were used for the cDNA synthesis. The integrity of isolated RNA was also checked on 1.2% agarose gel. cDNA was synthesized using the RevertAid™ H minus First Strand cDNA synthesis kit (Thermo fisher Scientific, Waltham, MA, USA), following the manufacturer’s protocol, and its quality was checked with Qubit^TM^ 2.0 Fluorometer (Invitrogen, UK). The transcript-specific oligos were used for the RT-PCR amplification using 2x PCR master mix (Promega, Madison, UK), and other steps were followed as mentioned in our earlier publication [6]. The amplified product was loaded onto 1% agarose gel, and an amplicon of ~1.2 kb was observed, which was further cloned in pGEM^®^-T Easyvector (Promega, Madison, UK) and transformed in *E. coli* strain DH5α competent cells, following the standard protocol [39], and sequenced using Sanger’s di-deoxy method.

### 4.4. In Silico Characterization of the Cloned HSF Gene

The cloned *HSF* gene was characterized for its homology using the BLASTn and BLASTp tools of the National Center for Biotechnology Information (NCBI; https://blast.ncbi.nlm.nih.gov/) (accessed on 18 December 2022). The nucleotide sequence was submitted to NCBI GenBank (http://www.ncbi.nlm.nih.gov/genbank/) (accessed on 15 January 2023). Translated sequence of the cloned *HSF* gene was predicted using Expasy tool (http://expasy.org/tools/) (accessed on 4 March 2023); open reading frame (ORF) was predicted using ORF Finder (http://www.ncbi.nlm.nih.gov/projects/gorf/) (accessed on 4 March 2023). The conserved domain (CD) of the gene was searched with CD search tool of NCBI (http://www.ncbi.nlm.nih.gov/Structure/cdd/wrpsb.cgi) (accessed on 4 March 2023). For multiple sequence alignment, amino acid sequences of HSFA2h and other closely related sequences were aligned using ClustalW2 alignment tool in order to analyze the sequence-specific variations, a phylogenetic tree was constructed using neighborhood joining method with boot strap values in CLUSTALX program by using full-length protein sequences of different closely related HSFs, as reported earlier. The target gene of candidate HSF, i.e., (*HSFA2h*), was predicted by Nsite Ver. 6.2014 (http://www.softberry.com/berry.phtml?topic=nsite&group=programs&subgroup=promoter) (accessed on 12 January 2023)**.**

### 4.5. Plasmid Construction of HSFA2h and Mobilization in Arabidopsis

The full-length wheat *HSFA2h* sequence was amplified using the Fp_HSFA2h-pRI101 and Rp_HSFA2h-pRI101 primers (Table 1). 

The amplified cDNA and pRI101-An vector (from Clontech) were restricted with *Nde*I and *Kpn*I restriction enzymes, gel purified, and ligated with T_4_ ligase enzyme (NEB) in order to clone the *HSFA2h* into the plant expression vector pRI 101-AN under the regulation of the cauliflower mosaic virus (*CaMV*)-35S constitutive promoter. The recombinant plasmids were mobilized into *Agrobacterium tumefaciens* EHA105 and further used to transform *A. thaliana* ecotype Columbia by the floral dip method [39]. Antibiotic-resistant transformed lines were further validated through qRT-PCR and Northern blot analyses. Homozygous T3 lines were further characterized for their thermotolerance using different biochemical and molecular parameters under HS. 

### 4.6. Southern Blot Analysis to Confirm the Transgenic Plants

Southern blot analysis was carried out on PCR-positive transgenic Arabidopsis plants at T_3_ stage. Genomic DNA was isolated from the transgenic Arabidopsis plant leaves by cetyl-tri-methyl ammonium bromide (CTAB) method [40]. Sixteen micrograms of genomic DNA were digested with the restriction endonucleases *Not*I (NEB, Ipswich, MA, USA) overnight at 37 °C and resolved on 0.8% agarose gel (15 V for 12 h). The resolved DNA was blotted onto a piece of nylon membrane (Hybond-N^+^, Amersham Biosciences, Uppsala, Sweden) using iBlot™ (Invitrogen, UK). DNA on the blot was denatured with an alkaline buffer (0.5 N NaOH, 1.5 M NaCl) for 2 min, soaked in a neutralizing buffer (0.5 M Tris HCl [pH 7.5], 1.5 M NaCl) for 2 min, and the membrane was UV-cross-linked and baked at 80 °C for 1 h. The baked membrane was pre-hybridized in a pre-hybridization buffer [0.5% sodium dodecyl sulfate (SDS), 6× SSC, 5× Denhardt’s solution] containing 100 μg of salmon sperm DNA (Pharmacia, Uppsala, Sweden) per mL at 65 °C for 4 h. The probe (*HSFA2h* fragment of ~600 bp generated after the restriction of the gene) was labeled with 50 μCi [α−^32^p] dCTP using DecaLabel DNA Labelling Kit (Thermo Fisher Scientific, Fermentas, Altrincham, UK). The labeled probe was used for the hybridization of the membrane at 65 °C for 16 h inside the hybridizer. The hybridized membrane was washed twice with 0.1% SDS in 2 × SSC (150 mmol/L NaCl, 15 mmol/L sodium citrate) solution for 10 min at room temperature, and then twice with 0.1% SDS in 0.2 × SSC solution for 20 min at 65 °C. An X-ray film was exposed to the air-dried radio-labeled membrane, and the signals were quantified using automatic X-ray developer machine. The appearance of the blot was captured using Gel Doc Easy (Bio Rad, Watford, Hertfordshire, UK).

### 4.7. Northern Blot Analysis to Characterize the Expression of Wheat HSFA2h in Transgenic Arabidopsis

Expression analysis of cloned *HSFA2h* gene was carried out in Southern positive transgenic Arabidopsis at T_3_ stage through Northern blotting. Total RNA was isolated from the collected samples by the Trizol method (Invitrogen, UK) and quantified with Qubit™ 2.0 Fluorometer (Invitrogen, UK); the integrity was verified on 1.2% agarose gel. For Northern blot analysis, 6 μg of total RNA was loaded onto 1.2% formaldehyde agarose gel and ran at 45 V for 2 h. The resolved RNA was blotted onto nylon membrane using iBlotter (Invitrogen, UK). The membrane was UV-cross-linked and baked at 80 °C for 1 h. Further, the membrane was pre-hybridized in a hybridization buffer (1% SDS, 1.5 M NaCl, 10% dextran sulfate) containing 100 μg of salmon sperm DNA (Pharmacia, Uppsala, Sweden) per mL at 65 °C for 4 h. *HSFA2h* DNA was labeled with α-[^32^P]-dCTP (BRIT, Bhabha Atomic Research Centre, India) and was used as a probe for the hybridization at 65 °C for 16 h. The membrane was washed twice with 0.1% SDS in 2 × SSC (150 mmol/L NaCl, 15 mmol/L sodium citrate) solution for 15 min, and then twice with 0.1% SDS in 0.2 × SSC solution for 15 min at 50 °C. An X-ray film was exposed to the air-dried radio-labeled membrane, and the signals were quantified using automatic X-ray developer.

### 4.8. Validation of Transgenic Arabidopsis through Quantitative Real-Time PCR (qRT-PCR)

Total RNA was isolated from the leaves of control and HS-treated transgenic Arabidopsis plants at T_3_ stage by Trizol method (Invitrogen, UK) and was quantified using Qubit™ 2.0 Fluorometer (Invitrogen, UK). First-strand cDNA synthesis was performed using oligo dT primers and Superscript II reverse transcriptase (Invitrogen, UK) according to the manufacturer’s instructions. First-strand cDNA was diluted to a final concentration of 100 ng μL^−1^ and real-time PCR was carried out, as described by Kumar et al. (2013). The primers for qRT-PCR were designed using the Gene Fischer primer designing software (http://bibiserv.techfak.uni-bielefeld.de/genefisher2/; accessed on 15 January 2023; Table 1). Three biological and technical replicates were used for the expression analysis. The expression levels of Arabidopsis β-*actin* gene (accession no. U41998.1) were used for normalizing the Ct value. The comparative C_t_ (2^−ΔΔCt^) method was used to calculate the relative fold expression of *HSFA2h* and its target gene *HSP18.2* [41].

### 4.9. Biochemical Screening of HSFA2h Expressing Transgenic Arabidopsis for Thermotolerance

#### 4.9.1. Estimation of Total Antioxidant Capacity (TAC)

Leaves of control and HS-treated samples collected from transgenic Arabidopsis at T_3_ stages and wild-type plants were used for the TAC estimation. TAC was assayed in the fresh leaves, as described by Benzie and Strain [42]. This method is based on the reduction of a ferric-tripyridyltriazine complex to its ferrous-colored form in the presence of antioxidants. The antioxidant capacity was expressed as the concentration of antioxidants having a ferric-reducing ability equivalent to that of 1 mmol L^−1^ FeSO_4_. 

#### 4.9.2. Antioxidant Enzyme Assay in Transgenic Arabidopsis

Fresh leaf material (1 g) was crushed in 5 mL of ice-cold 50 mM potassium phosphate buffer (pH 7.0) containing 2 mM sodium-EDTA and 1% (*w*/*v*) polyvinyl-pyrrolidone (PVP). The homogenates were centrifuged at 10,000 g (4 °C) for 10 min. The tissue extracts were used for the quantification of soluble protein content by using Bradford method [43]. 

The activity of guaiacol peroxidase (GPX) was determined following the method of Mika and Luthje [44] with slight modifications. The oxidation of guaiacol into tetraguaiacol was estimated by measuring the absorbance at 470 nm against the reagent blank using extinction coefficient of 26.6 mM^−1^ cm^−1^. The activity of catalase enzyme was measured as described by Rucinska et al. [45]. For assaying CAT activity, the decomposition of H_2_O_2_ was followed by decline in the absorbance at 240 nm. CAT activity was determined by following the consumption of H_2_O_2_ (extinction coefficient, 39.4 mM^−1^ cm^−1^) at 240 nm over a 3 min interval. Superoxide dismutase (SOD) activity was determined in crude extract by measuring its ability to inhibit the photochemical reduction of nitro-blue tetrazolium (NBT) in the presence of riboflavin in light [46]. One unit of enzyme activity was determined as the amount of enzyme needed for the inhibition of 50% NBT reduction rate by monitoring absorbance at 560 nm with spectrophotometer.

### 4.10. Physiological Characterization of HSFA2h-Expressing Transgenic Arabidopsis for Thermotolerance

#### 4.10.1. Infra-Red Gas Analyzer Analysis

The WT and HSFA2h overexpressing transgenic lines under control and HS treatment during the inflorescence stage were subjected to infra-red gas analyzer (IRGA) for analyzing the effect of HS on photosynthesis (LiCor 6400, LiCor Inc., Lincoln, NE, USA), and the observations were recorded following Long and Bernacchi [47]. Fully rosette leaves were used for the IRGA analysis, and the readings were taken in triplicate between 11 a.m. and 12 p.m. Photosynthesis was measured at constant saturating light of 1500 μmol m^−2^ s^−1^. Measurements of leaf R_dark_ were made on dark-adapted leaves after 30 min of dark adaptation to achieve steady-state R_dark_.

#### 4.10.2. Measurements of Chlorophyll Content

Chlorophyll content was estimated by the non-maceration method according to Hiscox and Israelstam [48]. Leaf samples (100 mg) from control and HS-treated plants in three different replicates were incubated in 20 mL of DMSO at 65 °C for 4 h in dark. Absorbance was recorded at 645 and 663 nm in a UV–Vis spectrophotometer (Thermo scientific, Evolution 220), and chlorophyll content was expressed as µmole g^−1^ fresh weight.

### 4.11. Statistical Analysis

We used completely randomized design for the experiment layout. The experiment was conducted with uppermost fully expanded leaves (in triplicate). One-way analysis of variance (one-way ANOVA) was used for the data analysis.

## 5. Conclusions

We identified ~37 transcripts showing homology with heat-responsive TFs from wheat through a de novo transcriptomic approach. Further, we cloned a putative heat-responsive TF, named *HSFA2h*, from wheat under HS and observed sHSPs as its potential targets. Expression analysis of *HSFA2h* and its target (*HSP17*) showed an abundance of transcripts in the leaf tissue of thermotolerant wheat *cv*. HD2985 under HS as compared to control. We developed transgenic Arabidopsis lines with the pRI101-HSFA2h construct and validated the integration of the TF gene through Southern and Northern blotting. The target gene of *HSFA2h* identified in Arabidopsis was *HSP18.2.* The expression of *HSP18.2* was observed to be regulated by *HSFA2h*, and cumulatively, both *HSFA2h* and *HSP18.2* expression help the plant develop tolerance against HS. Here, we established a positive correlation between the expression of *HSFA2h* and *HSP18.2;* the expression of s*HSP18.2* was observed at its maximum in transgenic plants under HS as compared to WT plants. The physio-biochemical traits linked with HS tolerance were better observed in *HSFA2h*-overexpressing transgenic lines than in the wild-type plants. Findings in the present investigation open a new dimension in the mechanisms of HS tolerance in wheat through transcriptional regulation of sHSPs. There is a need to validate the present findings in other crop plants and to enrich the genetic resources in terms of *HSFA2h* TF and sHSPs in order to understand the mechanisms underlying HS tolerance in agriculturally important crop plants. 

## Figures and Tables

**Table 1:** List of primers used for the cloning and expression analysis of HSFs and sHSP through quantitative real-time PCR (qRT-PCR).

* Primer ID	Oligo Sequence (5′-3′)	Tm (°C)
Fp_HSFA2h	ATGGACCCGGTGCCGAGTCTG	58 °C
Rp_HSFA2h	TAGGTTGAGAGGGTTGGGCTATTTG	58 °C
FpHSFA2h-pRI101	GGAATTCCATATGGACCCGGTGCCGAGTCTG	62 °C
RpHSFA2h-pRI101	GGGGTACCCTAGGTTGAGAGGGTTGGGCTATTTG	63.2 °C
qFp_HSFA2h	ACAGAGCCACAGGATTTTGG	58 °C
qRp_HSFA2h	TGAGAGGGTTGGGCTATTTG	58 °C
qFp_HSP17	GAGGGAGGAGAAGGAGGAC	57.7 °C
qRp_HSP17	TCGCTACTCTCTGCTTCGAT	57.9 °C
qFp_HSP18.2	CTGCAGATTAGCGGAGAGAG	58 °C
qRp_HSP18.2	ACAACCGTAAGCACACCATT	58 °C
qFp_AT-actin-2	AAGCTGGGGTTTTATGAATGG	58 °C
qRp_AT-actin-2	GGGACTAAAACGCAAAACGA	58 °C
qFp_β-Actin-F	GCG GTCGAACAACTGGTATT	63.7 °C
qFp_ β-Actin-F	GGT CCAAACGAAGGATAGCA	63.8 °C

* Fp—forward primer; Rp—reverse primer; q—quantitative; AT—*Arabidopsis thaliana*.

## Data Availability

The datasets generated during and/or analyzed during the current study are available in the NCBI repository [https://www.ncbi.nlm.nih.gov/nuccore/KP257297.1 (accessed on 4 March 2023)].

## Supplementary Materials

The following supporting information can be downloaded at: https://www.mdpi.com/article/10.3390/plants12203598/s1, Figure S1: Phylogenetic analysis of the relationships between *Triticum aestivum HSFA2h* gene and other species; the multiple alignment was performed using ClustalW2 and the dendrogram was built using neighborhood joining method with boot strap values in CLUSTALX program; Figure S2: The binary vector construct of wheat *HSFA2h* developed in pRI101-AN vector under 35S promoter; Table S1: Identification of novel heat-responsive transcription factors (TFs) and their chromosomal localization in wheat using de novo transcriptomic approach.
