# Supplementary material for: Transcriptional Regulation of Small Heat Shock Protein 17 (sHSP-17) by Triticum aestivum HSFA2h Transcription Factor Confers Tolerance in Arabidopsis under Heat Stress
Source: Plants (Basel). 2023 Oct 17;12(20):3598. doi: 10.3390/plants12203598 (PMC10609734; doi:10.3390/plants12203598)
Supplement: Supplementary file 1 [file plants-12-03598-s001.zip › Table S1.pdf]

Table S1. Identification of novel heat-responsive transcription factors (TFs) and their chromosomal localization in wheat using *de novo* transcriptomic approach.

Transcript IDs	Chromosomal Localization	Notation
HD2985_Stress_transcript_9649	dna:scaffold scaffold:IWGSP1:IWGSC_CSS_4DL_scaff_4DL_14373906:1:4475:1	HSHD2985_Stress_TF a-1b
HD2985_Control_transcript_12551	dna:scaffold scaffold:IWGSP1:IWGSC_CSS_2AL_scaff_6370272:1:4961:1	heat shock protein binding
HD2985_Stress_transcript_10840	dna:scaffold scaffold:IWGSP1:IWGSC_CSS_3DS_scaff_2560988:1:5582:1	heat-shock protein binding
HD2985_Stress_transcript_12230	dna:scaffold scaffold:IWGSP1:IWGSC_CSS_2AL_scaff_6439261:1:7602:1	heat shock factor protein 4
HD2985_Stress_transcript_12337	dna:scaffold scaffold:IWGSP1:IWGSC_CSS_1AL_scaff_3975644:1:3913:1	heat shock protein 101 binding
HD2985_Stress_transcript_13053	dna:scaffold scaffold:IWGSP1:IWGSC_CSS_7BS_scaff_3143055:1:7681:1	heat-shock protein binding
HD2985_Stress_transcript_16267	dna:scaffold scaffold:IWGSP1:IWGSC_CSS_7AS_scaff_4217917:1:8113:1	heat-shock protein binding
HD2985_Stress_transcript_16474	dna:scaffold scaffold:IWGSP1:IWGSC_CSS_4AS_scaff_5934783:1:12253:1	hsf-type dna-binding domain
HD2985_Stress_transcript_16876	dna:scaffold scaffold:IWGSP1:IWGSC_CSS_5DL_scaff_4598213:1:13044:1	heat shock factor protein 4
HD2985_Stress_transcript_17958	dna:scaffold scaffold:IWGSP1:IWGSC_CSS_7AS_scaff_4217917:1:8113:1	heat-shock protein binding
HD2985_Stress_transcript_19401	dna:scaffold scaffold:IWGSP1:IWGSC_CSS_7AS_scaff_4217917:1:8113:1	heat-shock protein binding
HD2985_Stress_transcript_23590	dna:scaffold scaffold:IWGSP1:IWGSC_CSS_7AS_scaff_4217917:1:8113:1	heat-shock protein binding
HD2985_Stress_transcript_411	dna:scaffold scaffold:IWGSP1:IWGSC_CSS_1DL_scaff_2241610:1:3486:1	heat shock protein 101 binding
HD2985_Stress_transcript_461	dna:scaffold scaffold:IWGSP1:IWGSC_CSS_1BL_scaff_3852546:1:4535:1	dnaj heat shock family protein
HD2985_Stress_transcript_733	dna:scaffold scaffold:IWGSP1:IWGSC_CSS_1BL_scaff_3852546:1:4535:1	dnaj heat shock family protein
HD2985_Stress_transcript_7730	dna:scaffold scaffold:IWGSP1:IWGSC_CSS_4DL_scaff_4DL_14373906:1:4475:1	HS HD2985_Stress_TF factor a-1b
HD2985_Stress_transcript_898	dna:scaffold scaffold:IWGSP1:IWGSC_CSS_7BS_scaff_3143057:1:9636:1	heat-shock protein binding
HD2985_Stress_transcript_9315	dna:scaffold scaffold:IWGSP1:IWGSC_CSS_4DL_scaff_4DL_14373906:1:4475:1	HS HD2985_Stress_TF a-1b
HD2985_Stress_transcript_9632	dna:scaffold scaffold:IWGSP1:IWGSC_CSS_4AS_scaff_5936883:1:19007:1	HS HD2985_Stress_TF a-1b
HD2985_Stress_transcript_7291	dna:scaffold scaffold:IWGSP1:IWGSC_CSS_1AL_scaff_3922902:1:6366:1	dnaj heat shock factor protein
HD2329_Stress_transcript_621	dna:scaffold scaffold:IWGSP1:IWGSC_CSS_4AS_scaff_5927411:1:24094:1	heat-shock protein 70t-2 binding
HD2329_Stress_transcript_1085	dna:scaffold scaffold:IWGSP1:IWGSC_CSS_7BS_scaff_3143055:1:7681:1	heat-shock protein binding
HD2329_Stress_transcript_1177	dna:scaffold scaffold:IWGSP1:IWGSC_CSS_7BS_scaff_3082537:1:7804:1	heat-shock protein binding
HD2329_Stress_transcript_5774	dna:scaffold scaffold:IWGSP1:IWGSC_CSS_2AL_scaff_6370272:1:4961:1	heat shock protein binding
HD2329_Stress_transcript_7756	dna:scaffold scaffold:IWGSP1:IWGSC_CSS_4BL_scaff_6990050:1:3750:1	HS HD2329_Stress_TF a-1b
HD2329_Stress_transcript_8502	dna:scaffold scaffold:IWGSP1:IWGSC_CSS_2BL_scaff_8050210:1:14690:1	HD2329_Stress_TF rf2b-like
HD2329_Stress_transcript_10598	dna:scaffold scaffold:IWGSP1:IWGSC_CSS_3DS_scaff_2560988:1:5582:1	heat-shock protein binding
HD2329_Stress_transcript_10836	dna:scaffold scaffold:IWGSP1:IWGSC_CSS_3B_scaff_10495529:1:5234:1	heat-shock protein binding
HD2329_Stress_transcript_16627	dna:scaffold scaffold:IWGSP1:IWGSC_CSS_2BL_scaff_8037467:1:10372:1	heat shock factor protein 4
HD2329_Stress_transcript_18200	dna:scaffold scaffold:IWGSP1:IWGSC_CSS_5DL_scaff_4598213:1:13044:1	heat shock factor
HD2329_Stress_transcript_22436	dna:scaffold scaffold:IWGSP1:IWGSC_CSS_2DL_scaff_9898261:1:4179:1	heat shock factor protein 4
HD2329_Stress_transcript_27031	dna:scaffold scaffold:IWGSP1:IWGSC_CSS_7AS_scaff_4217917:1:8113:1	heat-shock protein binding
HD2329_Control_transcript_1428	dna:scaffold scaffold:IWGSP1:IWGSC_CSS_2DS_scaff_5389432:1:16313:1	heat shock protein 90 binding
HD2329_Control_transcript_2715	dna:scaffold scaffold:IWGSP1:IWGSC_CSS_1BL_scaff_3852546:1:4535:1	heat shock protein binding
HD2329_Control_transcript_6183	dna:scaffold scaffold:IWGSP1:IWGSC_CSS_7BS_scaff_3143055:1:7681:1	heat-shock protein binding
HD2329_Control_transcript_8846	dna:scaffold scaffold:IWGSP1:IWGSC_CSS_1DL_scaff_2241610:1:3486:1	heat shock protein 101 binding
HD2329_Control_transcript_12353	dna:scaffold scaffold:IWGSP1:IWGSC_CSS_4DL_scaff_4DL_14373906:1:4475:1	HS HD2329_Control_TF a-1b
