# Supplementary figures and images for: Transcriptional Regulation of Small Heat Shock Protein 17 (sHSP-17) by Triticum aestivum HSFA2h Transcription Factor Confers Tolerance in Arabidopsis under Heat Stress
Source: Plants (Basel). 2023 Oct 17;12(20):3598. doi: 10.3390/plants12203598 (PMC10609734; doi:10.3390/plants12203598)

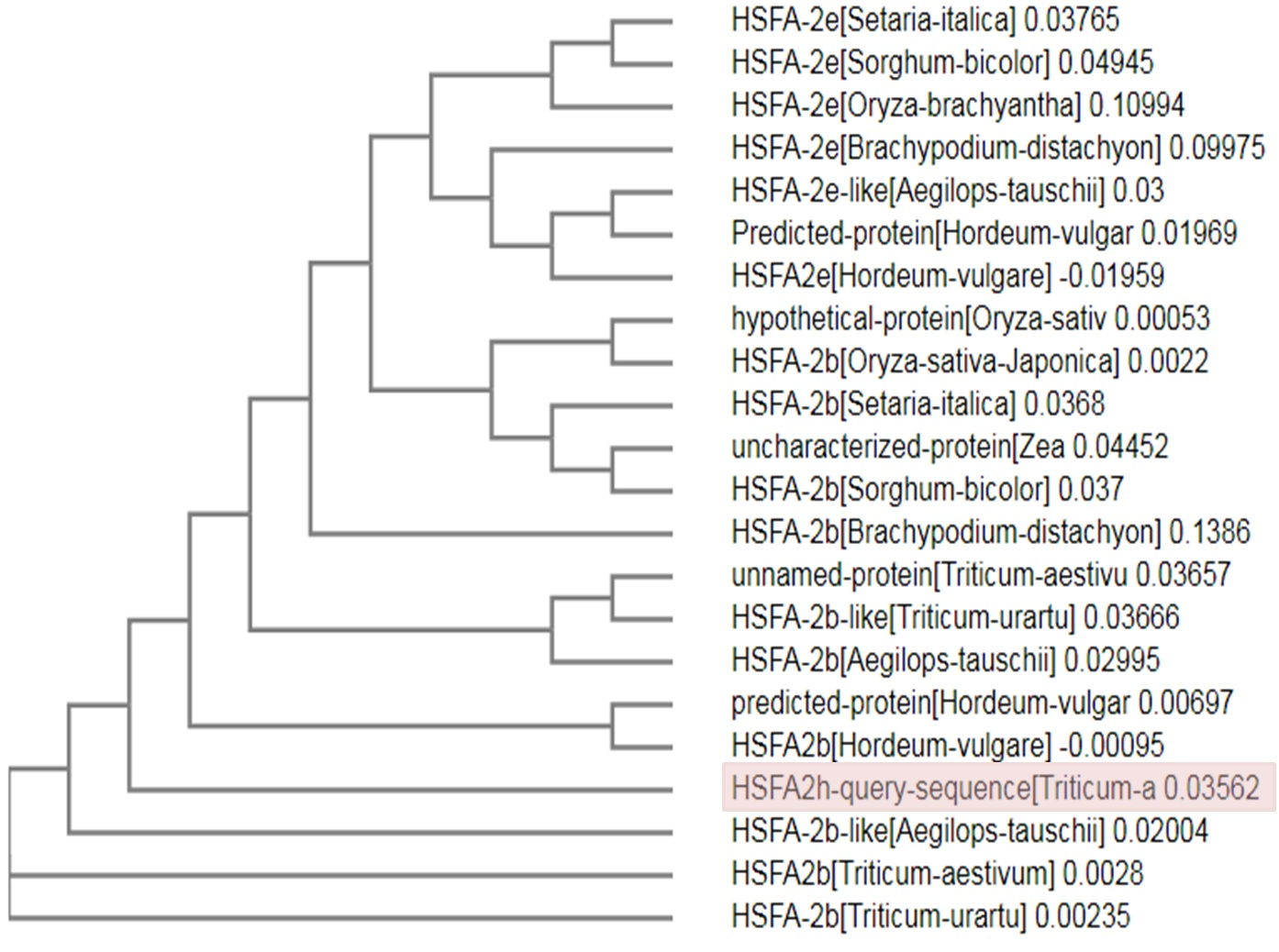

Supplement: Supplementary file 1 [file plants-12-03598-s001.zip › Figure S1.tif]

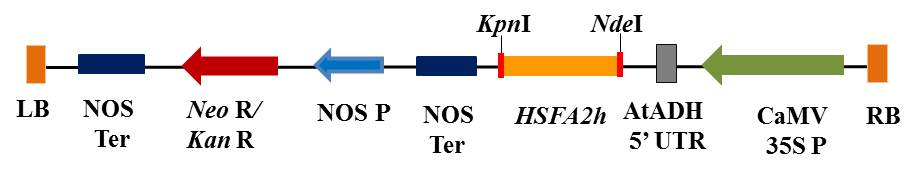

Supplement: Supplementary file 1 [file plants-12-03598-s001.zip › Figure S2.tif]
